# Vibrational and Acoustical Characteristics of Ear Pinna Simulators That Differ in Hardness

**DOI:** 10.3390/audiolres11030030

**Published:** 2021-07-01

**Authors:** Ryota Shimokura, Tadashi Nishimura, Hiroshi Hosoi

**Affiliations:** 1 Department of Systems Science, Graduate School of Engineering Science, Osaka University, Osaka 560-8531, Japan; 2 Department of Otolaryngology—Head & Neck Surgery, Nara Medical University, Nara 634-8521, Japan; t-nishim@naramed-u.ac.jp; 3 Medicine-Based Town Institute, Nara Medical University, Nara 634-8521, Japan; hosoi@naramed-u.ac.jp

**Keywords:** cartilage conduction, pinna simulator, head-and-torso simulator, hearing aid, vibration acceleration level, sound pressure level

## Abstract

Because cartilage conduction—the transmission of sound via the aural cartilage—has different auditory pathways from well-known air and bone conduction, how the output volume in the external auditory canal is stimulated remains unknown. To develop a simulator approximating the conduction of sound in ear cartilage, the vibrations of the pinna and sound in the external auditory canal were measured using pinna simulators made of silicon rubbers of different hardness (A40, A20, A10, A5, A0) as measured by a durometer. The same procedure, as well as a current calibration method for air conduction devices, was applied to an existing pinna simulator, the Head and Torso Simulator (hardness A5). The levels for vibration acceleration and sound pressure from these pinna simulators show spectral peaks at dominant frequencies (below 1.5 kHz) for the conduction of sound in cartilage. These peaks were likely to move to lower frequencies as hardness decreases. On approaching the hardness of actual aural cartilage (A10 to A20), the simulated levels for vibration acceleration and sound pressure approximated the measurements of human ears. The adjustment of the hardness used in pinna simulators is an important factor in simulating accurately the conduction of sound in cartilage.

## 1. Introduction

**Figure 1:**
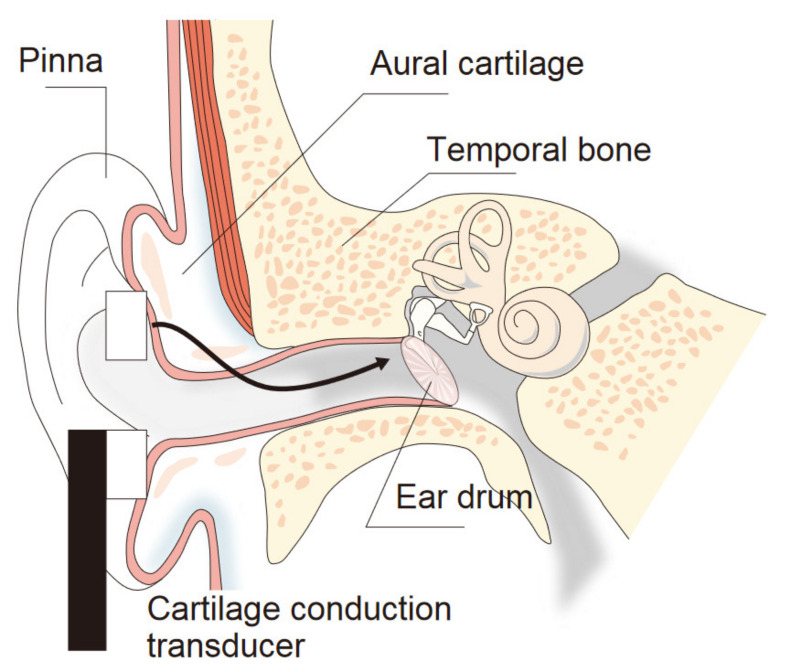
Cartilage conduction pathway for users without hearing impairments.

Aural cartilage gives form to the pinna and the exterior half of the external auditory canal. If the aural cartilage vibrates, sound can be clearly heard [1,2]. This phenomenon is termed *cartilage conduction*, and hearing aids based on cartilage conduction [2,3,4,5] have been marketed in Japan since November 2017. When a small transducer is fixed at the entrance of the ear canal, it can generate sound via the aural cartilage into the external auditory canal (Figure 1) [6,7,8,9]. That is, this cartilage acts as a diaphragm and the transducer functions as a voice coil of a loudspeaker. Distinct from bone-conduction hearing aids, the cartilage conduction transducer is small and light, and contact pressure on the cartilage is very low because the cartilage is light and vibrates more easily than heavy skull bone. Indeed, the vibrations propagating through skull bone are small enough that their contribution to hearing can be ignored when the cartilage is stimulated [5].

**Figure 2:**
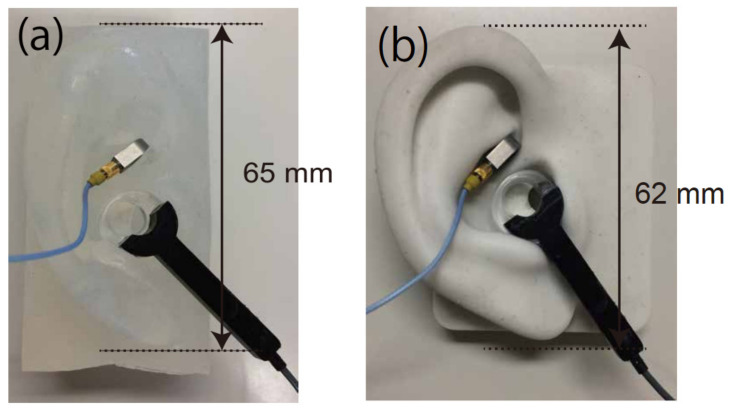
Position of the cartilage conduction transducer and accelerometer of (**a**) pinna simulator made for this study and (**b**) pinna simulator of the HATS.

Although cartilage conduction hearing aids can decrease hearing thresholds, especially of users with aural atresia, otorrhea, and microtia [10,11], they are not covered by insurance for the physically handicapped persons in Japan, because output volumes have not been standardized. Because the transmission pathway of cartilage conduction differs from either air or bone conduction (Figure 1), their calibration methods, standardized in the International Organization for Standardization [12,13], do not apply to cartilage conduction-based devices. For example, for air conduction, the sound pressure at the ear drum can be simulated using an ear simulator [14] embedded in a head-and-torso simulator (HATS) [15]. However, in terms of the cartilage sound, the HATS cannot output the same sound pressure as actual measurements from human ears, especially in the low-frequency range below 1.5 kHz [16]. Nevertheless, a soft polyurethane pipe can simulate the aural cartilage of the external auditory canal and a skull bone model can produce the sound pressure in agreement with data from human ears in this low-frequency range [13]. In that study, a ring-shaped transducer (same type in Figure 2) adhered to the pipe and was worn by seven participants, and the sound pressures in the polyurethane pipe and human canal were measured by a probe microphone, which was inserted in the pipe and canal to a point 15 mm from each entrance. The polyurethane resin was designed to simulate the elasticity of human skin (human skin gel, Exseal Corporation, Mino, Japan). In contrast, the pinna simulator of the HATS (Type 4128; Brüel & Kjaer, Naerum, Denmark) is embedded as a unit in a silicon rubber base (width × height × thickness: 50 × 60 × 10 mm^3^). The hardness (Shore 00 35 or Shore A5 from durometer measurements) of the pinna simulator is lower than that of the aural cartilage of humans. In the low-frequency range, simulation results are likely to disagree because of this mismatch in hardness for the pinna simulator of HATS.

Therefore, the propagating vibration in aural cartilage and generating sound in the external auditory canal were measured in our study of five ear simulators, each made of silicon rubber of a different hardness. A hardness of aural cartilage was measured at the tragus beforehand (see Section 2.3), and the hardness of the silicon rubbers were determined to diverge higher and lower than it. For comparison, the same measurements were recorded for the pinna simulator of the HATS. Different spectral characteristics were observed that depended on the hardness of each silicon rubber.

## 2. Method

### 2.1. Pinna Simulators

**Figure 3:**
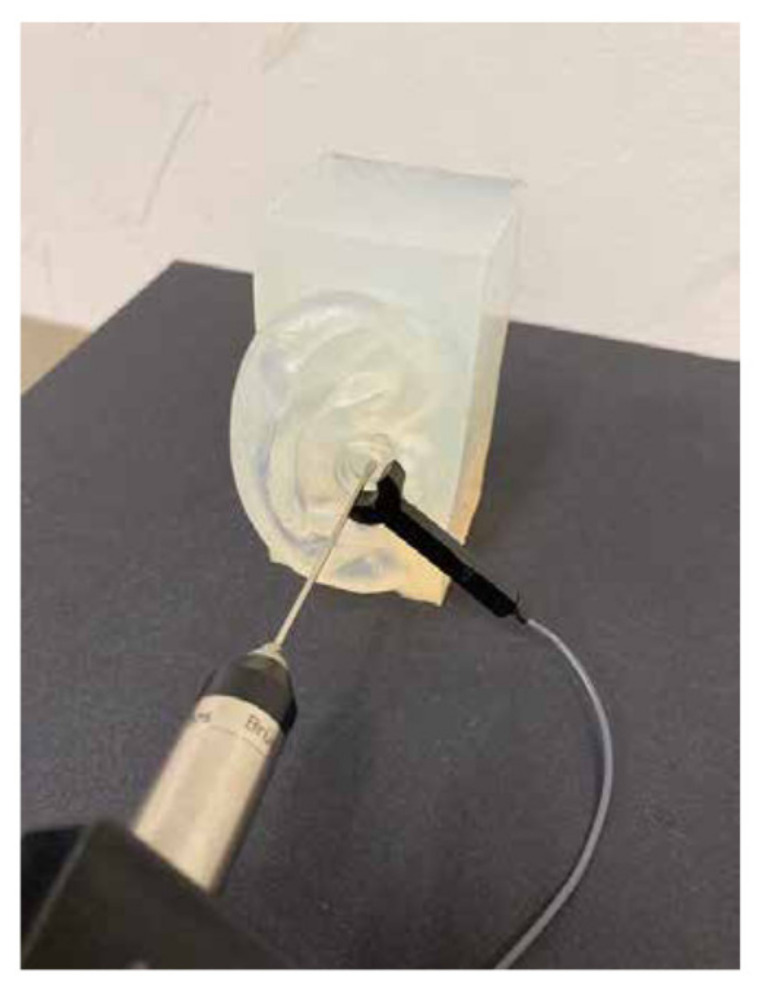
Position of the cartilage conduction transducer and probe microphone of pinna simulator.

Various silicon rubbers were molded into the shape of a human pinna and embedded in a base of width × height × thickness: 40 × 80 × 40 mm^3^ (Figure 2a and Figure 3). The external auditory canal (inner diameter: 10 mm; length: 35 mm) was excavated within the base. From durometer measurements, the hardness of the various silicon rubbers was classified into five classes: A0, A5, A10, A20, and A40. In addition to these pinna simulators, that of the HATS, with corresponding hardness A5, was used as a reference (Figure 2b).

### 2.2. Cartilage Conduction Transducer

In simulations, an annular transducer was used to induce cartilage conduction as in previous measurements [3,6,7,8,16] (see Figure 2 and Figure 3). The part worn is an acrylic ring (external diameter: 16 mm; internal diameter: 8 mm). The hole acts as an air vent to cancel effects from occlusions and enables cartilage conduction sound in the air canal to be recorded unmodified. The transducer is composed of a piezoelectric bimorph covered with elastic material. Although some resonance peaks appear in the vibrational output, the spectral characteristics are, on the whole, flat in the frequency range above 1 kHz [6]. The cartilage conduction transducer was fixed at the entrance of the external auditory canal between the concha wall and tragus (Figure 2).

### 2.3. Measurement Procedures for Vibration

The input signal for the transducer is a pure-tone train of frequency ranging from 125 Hz to 16 kHz in 1/12-octave steps. The tones were 1 s in duration, each followed by a silent interval of 0.5 s. The input level was 2.0 V. Vibration acceleration levels (VALs) were determined from the spectral peaks at the corresponding frequencies of the pure tones.

The propagating vibration of the pinna was measured using a subminiature piezotronics accelerometer (model 352A21; PCB Piezotronics, Depew, NY, USA) located on the cymba conchae without any adhesive bond (Figure 2) because a previous study reported that the spectral characteristics of the propagating vibration from the human pinna were not so different among conchae, tragus, and scaphoid fossa, especially below 1 kHz [3]. In addition to the five pinna simulators, the propagating vibration on a human ear (right ear of a male, 42 years old) was measured again for comparison of settings. The hardness of the aural cartilage at the tragus, obtained using a durometer (GS-719N; Teclock, Nagano, Japan), corresponded to A20 or A10. The measured signals were digitized for subsequent analysis with a sampling rate of 44.1 kHz and a 16-bit resolution (UA-101 analog-to-digital converter; Roland, Hamamatsu, Japan).

### 2.4. Measurement Procedure for Sound

Compared with the vibrational measurement, the procedure used to measure sound in the external auditory canal differed only in the signal receiver. The sound in the auditory canal of the pinna simulator was recorded using a calibrated probe microphone (type 4182; Brüel & Kjaer, Naerum, Denmark), which has a metallic probe tube (length: 100 mm, diameter: 1.24 mm) that allowed sound pressure to be measured in a narrow or enclosed space (Figure 3). The probe was inserted into the external auditory canal through the hole in the annular transducer, without touching it. The tip of the probe was extended 15 mm from the entrance of the auditory canal. The measurement position and procedures were the same as those reported in our previous study [16]. The sound recordings were performed in a soundproof chamber whose background noise was less than 30 dB. Sound pressure levels (SPLs) were determined from the spectral peak at each corresponding frequency of the pure tones.

The SPLs in the auditory canal for the HATS and human ears were extracted from our previous study [16], in which the same cartilage conduction transducer was used. While wearing the transducer (Figure 2b), the SPLs of the HATS were measured as sound passed through the artificial ear mounted on the HATS. The SPLs obtained from the auditory canal of seven participants (25–36 years old) were measured with the same probe microphone (Figure 3) [16]. The SPLs for both the HATS and human ears were comparable to those from current pinna simulators. Before digitizing, the sound output was calibrated using a conditioning amplifier (NEXUS; Brüel & Kjaer, Naerum, Denmark).

## 3. Results

**Figure 4:**
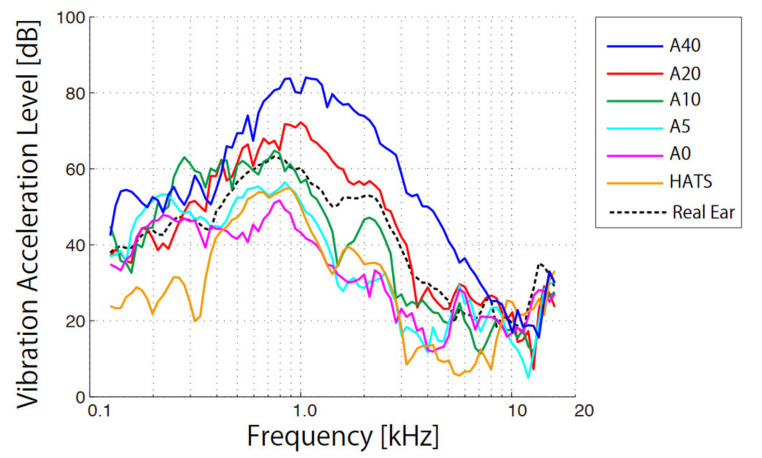
Vibration acceleration level (VAL) as a function of frequency for the various pinna simulators (A40, A20, A10, A5, A0, HATS) and human ear.

The VALs obtained from the different pinna simulators were compared with those of a human ear (Figure 4) over the same range of frequencies (<16 kHz). Each curve was determined from the average outputs from three measurements while wearing the accelerometer and transducer and on their removal. Although each line was uneven with small peaks and dips, it was smoothened if the measurement times increased. The standard deviation for each frequency was on average 4.8 dB for A40, 4.9 dB for A20, 5.3 dB for A10, 4.5 dB for A5, 6.1 dB for A0, 3.0 dB for HATS, and 4.9 dB for a human ear.

The spectral profiles showed a flat or upward trend, up to approximately 1 kHz, and typically decreased after reaching the edge frequency. The characteristics of the peak maxima were 84.0 dB (1059 Hz) for A40, 72.2 dB (1000 Hz) for A 20, 64.8 dB (749 Hz) for A10, 56.5 dB (841 Hz) for A5, 51.7 dB (793 Hz) for A0, 55.0 dB (793 Hz) for HATS, and 63.3 dB (749 Hz) for the human ear. Both peak values and corresponding frequencies decreased with the softening of the pinna hardness. The errors from the VALs of the human ear on average were 14.0 dB for A40, 5.3 dB for A20, 6.4 dB for A10, 9.0 dB for A5, 9.7 dB for A0, and 13.0 dB for HATS.

**Figure 5:**
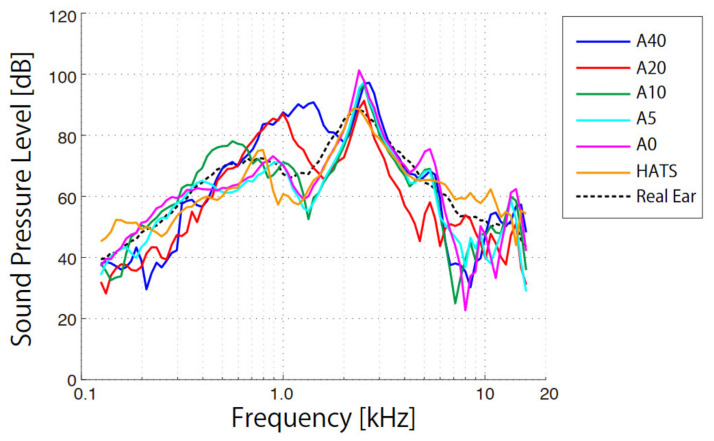
Sound pressure level (SPL) as a function of frequency for the various pinna simulators (A40, A20, A10, A5, A0, HATS) and human ear.

As for the VAL measurements, the SPLs of the different pinna simulators were measured (Figure 5), each curve being determined from the average outputs among three measurements, while wearing the probe microphone and transducer and on their removal, to assess reproducibility. The standard deviations for each frequency were on average 3.1 dB for A40, 4.7 dB for A20, 2.2 dB for A10, 3.3 dB for A5, and 2.0 dB for A0. The spectral profiles for the HATS and human ear (orange and dashed curves, respectively) are from our previous study [16], in which all measurement instruments and procedures were the same. The value at each frequency was also the average of three measurements obtained from the HATS and the auditory canal of the seven participants.

From the SPLs of the human ear (dashed curve), two clear spectral peaks were evident. The sharp peak at 2.5 kHz arose as a resonance occurring in the external auditory canal, whereas the broad peak at 800 Hz corresponded to sound generated from cartilage conduction [13]. The lower peak disappeared when the transducer was 7 mm distant from the aural cartilage (non-touching condition) [6]. The resonance peak at 2.5 kHz did not change for any of the five pinna simulators because the length of each auditory canal remained the same. However, the peaks arising from cartilage conduction from 700 Hz to 1.5 kHz moved to lower frequencies with the softening of the pinna hardness. The peaks were 90.8 dB (1414 Hz) for A40, 87.2 dB (1000 Hz) for A20, 78.1 dB (561 Hz) for A10, 71.2 dB (944 Hz) for A5, 73.2 dB (891 Hz) for A0, and 75.2 dB (794 Hz) for HATS. As for the vibrational characteristics, both the peak values and corresponding frequencies exhibited a decreasing trend with softening hardness. For the low-frequency range below 1.5 kHz, the errors from the SPLs of the human ear on average were 9.4 dB for A40, 8.3 dB for A20, 4.6 dB for A10, 4.1 dB for A5, 3.7 dB for A0, and 5.7 dB for HATS.

## 4. Discussion

Silicon rubber has a resonance frequency that depends on Young’s modulus (i.e., hardness). One procedure for the measurement of Young’s modulus is the dynamical resonance method, which determines Young’s modulus (*E* N/m^2^) by measuring the resonance frequency (*f* Hz) using

(1) $$E=0.9467\times {\left(\frac{l}{h}\right)}^{3}\times \frac{m}{w}\times f^{2}$$

here, the test sample has mass (*m* kg) and dimensions (length: (*l* m), width: (*w* m), height (*h* m)) [17]. Although this method applies to steel slabs, Young’s modulus of soft silicon rubber is difficult to quantify precisely [18], the resonance frequency being dependent on Young’s modulus and hardness.

In the same way, the maximum peaks of propagating vibrations through softer silicon-based pinna simulators were obtained at lower frequencies (Figure 4). Because the pinna simulator of the HATS has a similar hardness of A5, their spectral profiles (cyan and orange curves) are similar. The spectral profiles of the pinna simulators with A20 and A10 (red and green curves) also approximate that of a human ear (black, dashed curve); the averaged VAL errors were lowest for the human ear with hardness A20 and A10. From the hardness measurements obtained using the same durometer, the hardness of the human ear at the tragus corresponded to A20 or A10. These results suggest that vibration simulations of the aural cartilage are important when adjusting the hardness of human aural cartilage. The pinna simulator of the HATS was too soft compared with that for human cartilage and, therefore, in the lower-frequency range below 1.5 kHz. The measured SPLs obtained from the HATS did not agree with those from participants [16].

The results from vibration simulations reflected well the SPL values from the external auditory canal of the pinna simulators (Figure 5). The resonance peaks in cartilage conduction typically appear below 1.5 kHz and move to lower frequencies with the softening of the pinna hardness, similar to the peaks of the VAL curves. The frequencies of the peaks in the SPL curves correlated strongly with those of the VAL curves (*r* = 0.89, *p* < 0.01). When the external auditory canals of participants lying on their sides were filled with water, the hearing threshold of the cartilage conduction lowered at the instant when the water surface reached the aural cartilage [8]. This psycho-acoustical experiment indicated a transmission mechanism in which the vibrating cartilage generates sound in the external auditory canal. Previous physical measurements indicated that certain transducer positions vibrate the aural cartilage more effectively, making sound louder in the external auditory canal [19]. In physical measurements, the VALs in the cartilage and the SPLs in the canal were measured using several transducer positions in the canal entrance of participants, whereas the pinna simulators employed in this study showed a clearer relationship between VAL and SPL because the transducer and accelerometer positions were fixed.

In the lower-frequency range below 1.5 kHz, the peak frequency of the SPL for the human ear (749 Hz) was between that for A20 (1000 Hz) and A10 (561 Hz). Similar to the vibration results, the results agreed with the hardness of actual aural cartilage subjected to sound pressures of a similar spectral profile. While the hardness set for the pinna simulators for the HATS was too soft to simulate the vibration in a human aural cartilage, the artificial ear embedded in the HATS could precisely simulate the behavior in the higher-frequency range above 1.5 kHz [16]. In a future study, pinna simulators of differing hardness for the HATS are to be made, and the sound output will be compared with the measured SPLs from the ears of participants. Following the conclusions obtained from the abovementioned results, the simulated SPL is expected to agree with the measured data when the hardness of the pinna simulator approximates that of human aural cartilage.

### Limitation of the Study

In this study, the hardness of aural cartilage was measured by one participant (male, 42 years old) whose pinna did not have any disorder. However, it is possible that the hardness of aural cartilage may be varied according to the age, sex, and disorder, and the vibrational and acoustical characteristics of the cartilage conduction may be changed as the results. Further study is required to examine statistical analyses regarding the hardness.

## 5. Conclusions

Using five pinna simulators differing in hardness, vibrations at the pinna and sound in the external auditory canal were measured and compared with those of a human ear for the purpose of calibrating cartilage conduction of sound. From VAL and SPL curves, we found that the spectral characteristics for the pinna simulators approached those of the human ears when their hardness coincided. The simulation of cartilage conduction of sound using the HATS is possible if the hardness of the pinna simulator is adjusted to that of human aural cartilage.

## Data Availability

Not applicable.
